# A Survey on Proactive, Active and Passive Fault Diagnosis Protocols for WSNs: Network Operation Perspective

**DOI:** 10.3390/s18061787

**Published:** 2018-06-01

**Authors:** Amjad Mehmood, Nabil Alrajeh, Mithun Mukherjee, Salwani Abdullah, Houbing Song

**Affiliations:** 1 Institute of Information Technology, Kohat University of Science Technology, Kohat KP 26000, Pakistan; 2 Traffic Safety Technologies Chair, Urban Planning Department, College of Architecture and Planning, King Saud University, Riyadh 14511, Saudi Arabia; nabil@ksu.edu.sa; 3 Guangdong Provincial Key Lab of Petrochemical Equipment Fault Diagnosis, Guangdong University of Petrochemical Technology, Maoming 525000, China; m.mukherjee@ieee.org; 4 Faculty of Information Science and Technology (Bangi Campus), Universiti Kebangsaan Malaysia, Bangi Selangor 43600, Malaysia; salwani@ukm.edu.my; 5 Department of Electrical, Computer, Software, and Systems Engineering, Embry-Riddle Aeronautical University, Daytona Beach, FL 32114, USA; h.song@ieee.org

**Keywords:** fault diagnosis, proactive, active, passive, network operation, wireless sensor networks

## Abstract

Although wireless sensor networks (WSNs) have been the object of research focus for the past two decades, fault diagnosis in these networks has received little attention. This is an essential requirement for wireless networks, especially in WSNs, because of their ad-hoc nature, deployment requirements and resource limitations. Therefore, in this paper we survey fault diagnosis from the perspective of network operations. To the best of our knowledge, this is the first survey from such a perspective. We survey the proactive, active and passive fault diagnosis schemes that have appeared in the literature to date, accenting their advantages and limitations of each scheme. In addition to illuminating the details of past efforts, this survey also reveals new research challenges and strengthens our understanding of the field of fault diagnosis.

## 1. Introduction

**Figure 1:**
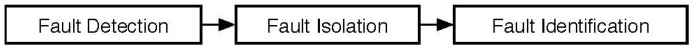
Fault diagnosis process.

Large-scale wireless sensor networks (WSNs) consist of hundreds or thousands of nodes that are wirelessly connected to sense the information from phenomena in their proximity. The sensed information is sent through a radio link to the central node, which is called the sink node, where it is used to make decisions. Over the past few decades, WSNs have attracted considerable attention because of their growing potential and positive impact on our daily life. These networks are normally application specific, such as, environmental surveillance [1,2], scientific observation [3,4], traffic monitoring [5]. Although ample research has been done on improving the reliability and robustness of these networks [6,7,8,9,10,11], network fault diagnosis has received little attention in the area of in situ network diagnosis for testing operational sensor networks under critical and crucial conditions. Fault diagnosis can help to extend the overall lifespan of WSNs, thereby enhancing the network’s ability to work for a longer period of time. Fault diagnosis can be separated into the following three processes: fault detection, fault isolation, and fault identification (see Figure 1). The fault detection determines whether a malfunction or fault has occurred in the system and it captures the time of its occurrence. Fault isolation determines the exact location of the fault. Finally, fault identification tracks the type, size and shape of the fault.

Owing to the ad-hoc nature of WSNs, it is difficult for managers or administrators to gain the requisite proximity to detect and manage each node after deployment, and then observe the inner structure and interactions occurring within the network from the outside. A WSN has the following characteristics that make it different from other networks: limited resources, dynamic topology, a large number of diverse types of nodes, hierarchical deployment methods, self-monitoring and deployment environment. Fault diagnosis of sensor networks is in great demand at present because it can help to make networks more energy efficient, reliable, robust and scalable. Diagnosing the network accurately and in a timely manner is important because otherwise the network would not able to achieve its intended objectives efficiently and faultlessly. For instance, GreenOrbs is a sensor network that consists of 300 nodes and is deployed in forests to monitor and assess activities [12,13]. On the network, sensor nodes receive information at regular time intervals from the forest to be monitored, and ensure that the nodes in the network operate faultlessly. Thus, fault diagnosis plays a vital role in GreenOrbs network monitoring because a fault can have a very large negative effect on the system’s efficiency and reliability [14,15,16,17,18,19,20].

In the literature, most of the research on WSN fault diagnosis has been carried out on proactive approaches in which the agents are implanted on sensor nodes to periodically report the information to the sink; for example, sensor node component failure information, neighboring node information, battery, and health status [21]. Zhao et al. [22] presented a proactive scheme in which each node collects residual energy and monitors parameters such as link loss rate and packet count. When this information is collected at each node, it is transmitted to the sink node for analysis. However, the proactive information gathering schemes generate a lot of traffic overhead during the information generation and their retrieval phases are usually fragile under high traffic loads. These approaches work like debugging and evaluation tools in a laboratory when deployed outside laboratory settings. Evaluation tools, such as IBM’s Tivoli [23,24] and Microsoft’s Operation Manager [25], have a considerable impact on offline debugging, where the behavior and scale of the network can be strictly controlled. These tools are effective for gathering large amounts of complex data through software agents in large-scale WSNs. However, the resource constraint WSNs bear a heavy energy cost because of the heavy calculations required to process complex data. In addition, these tools also limit the characteristics of self-organisation and dynamics of the network.

**Figure 2:**
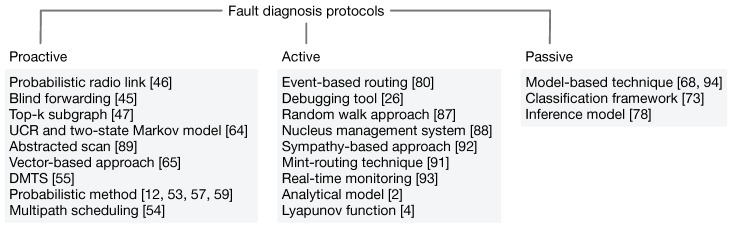
Fault diagnosis protocols.

The aforementioned diagnosis tools are not recommended for in situ diagnosis of deployed operational WSN because sensor nodes in these approaches continuously and vigorously generate a lot of traffic that ingests communication, computation and energy resources. Moreover, integrating these complex debugging tools with an application program at each sensor node creates difficulties in system implementation. Similarly, active fault diagnosis imposes a heavy traffic overhead on the network because a large amount of information is transferred regarding specific control commands or status information to the sink, such as in Sympathy [26] and Clairvoyant [27,28,29]. These active fault diagnosis approaches focused on determining and tracking the software faults of the sensor nodes, which tends to put the network under a heavy network traffic burden. To minimise the network traffic, passive fault diagnosis schemes have been suggested and practised as a solution (see Figure 2).

Passive fault diagnosis is motivated by the limitations of the previously described techniques. Recently, several ongoing sea monitoring projects [30,31,32] illustrate the importance of passive fault diagnosis. The project initiated a working prototype of a WSN consisting of tens of nodes floating on the surface of the sea to collect scientific information, such as depth, surrounding illumination, and pollution. In recent deployment tests, it has been observed that abnormal energy depletion would never occur in controlled laboratory settings, and such abnormalities only surface due to the utilisation of a multi-hop router component, which calculates the optimisation routing tree of the highly unstable environment of the sea and which also expects a high degree of delay and packet loss at the sink node. Moreover, faster and accurate determination of the root causes of a detected problem is required before further action can be taken, such as requesting reboot messages to some nodes or physically examining the suspicious links.

The main contributions of this paper are as follows:

- A survey of the techniques that are available for proactive, active and passive fault diagnosis approaches is presented.
- An overview of current research trends are discussed to address the issues in fault diagnosis. Moreover, a list of open research challenges are highlighted.
- Given that, to the best of our knowledge, there is no survey available on this topic from a network operation perspective, each aspect of fault diagnosis under proactive, active and passive schemes has been covered from the network operation point of view.

The paper is organized as follows. Section 2 presents an overview of fault diagnosis approaches for WSNs. Section 3 presents the related work on the three primary schemes mentioned above. In Section 4, we illustrate a fault detection algorithm. In Section 5, we introduce the commonly used terms and provide a brief explanation of the different schemes in order to present a general picture of the trends being followed in the implementation of each fault diagnosis scheme. In Section 5, we present our conclusions.

## 2. Overview of Fault Diagnosis for WSNs

### 2.1. Importance of Fault Diagnosis

Failures in WSNs are inevitable due to inhospitable environments, in which sensor nodes easily become faulty and unreliable. Therefore, fault diagnosis acts as a tool to improve the lifespan of a WSN and to effectively optimise bandwidth. For instance, if faulty nodes comprise part of the network activity, then erroneous data will be sent to the sink via intermediate nodes. The intermediate nodes use their limited energy in relaying that faulty data. If a large number of faulty sensor nodes become involved in the network, then this would decrease the overall network lifespan and will consume much bandwidth in transmitting erroneous or useless data. To address these issues, fault diagnosis in WSNs provides a list of all of the possible faulty sensor nodes in the network. By considering this list, future recovery becomes possible. The list includes faulty reading correction, replacing malfunctioning nodes with healthier ones, or simply isolating the faulty nodes from the network, provided that it has sufficient redundancy [33,34,35,36,37].

**Figure 3:**
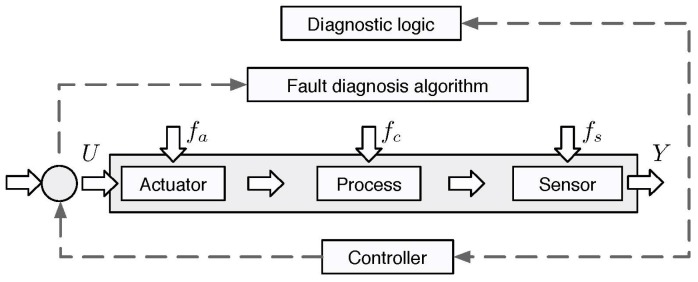
Fault diagnosis algorithm.

To address the issues of reliability and robustness, fault diagnosis is used to monitor, locate and identify hardware or software redundancy (also known as analytic redundancy). Hardware redundancy is most commonly achieved by deploying more hardware components that have the same signal. This duplication helps to make the diagnostic decision by using the methods of limit checking or majority voting. This redundancy is reliable, but it is also expensive. It increases physical weight and it also consumes more physical space. By considering the limitations of hardware redundancy, software or analytical redundancy was introduced in modern control theory in the 1980s (see Figure 3 for more details). For a controlled system subjected to actuator fault $f_{a}$, process/component fault $f_{b}$, and sensor fault $f_{s}$, the input *u* and output *y* are used to construct a fault diagnosis algorithm. This algorithm is used to check the consistency of system processes in real time by providing inputs and receiving outputs against predetermined information from a healthier system, and it then formulates a decision using diagnostic logic. Compared to hardware, software redundancy is very cost effective but it is more challenging to implement due to environmental noise, dynamic system complexity, control structures, and the inevitable modeling error. One of the most important functions of fault diagnosis is detecting network failures. Thus, it is important to understand network failures and their types.

### 2.2. Failure in WSNs

**Figure 4:**
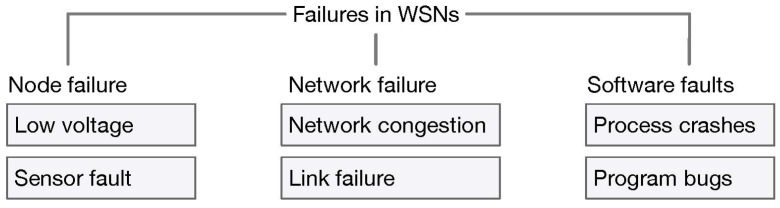
Types of failure in WSNs.

A fault is defined as an unusual change in or deviation from one or more characteristics of a system from standard, acceptable or usual conditions [38]. Sometimes, the failure is defined as the omission of occurrence or performance, failing to perform a duty or expected action, or failing to achieve prescribed goals or objectives [39]. Generally, three types of failures occur in WSNs: node, network and software failures (see Figure 4). Node failures occur in a network due to large numbers of nodes being deployed in harsh and/or inaccessible outdoor environments. Therefore, they can be destroyed or damaged easily. Moreover, any nodes in a network have limited energy that can also be depleted. Failures could also be caused by low power readings and sensor faults. Network failures could occur due to network devices or network services being in an abnormal state. This type of failure includes network congestion, link failure and looping. Software failure includes operating system process crashes and problems caused by program bugs. Although this type of failure commonly affects WSNs, the probability of occurrence is relatively small compared to other failures [40]. For a more detailed understanding of the fault diagnosis concept, a general fault model and diagnosis protocol must be first understood.

### 2.3. Fault Model and Diagnosis Protocol

WSNs consist of fragile nodes and uncertain links, which are the main sources of faults in networks. Fragile nodes’ failures occur due to physical damage or functional errors. Physical damage occurs, for example, when the nodes are dropped during the process of deployment, where they are damaged or functionally impaired when striking the ground, getting stuck in a tree, hitting physical objects or falling into water. Moreover, the functional errors occur when the collected data exhibit misbehavior. These are the most harmful errors in the WSN applications. Since sensor nodes are randomly deployed across a geographical area to monitor the environment, this randomness can affect the efficiency and performance of the diagnosis protocols. Taking these factors into account, whenever a fault diagnostic protocol is presented or analysed, we must consider the fragility of the nodes, link uncertainty and topological randomness [41,42]. The next section will describe the general procedure for performing fault detection in the network.

### 2.4. Fault Detection

It is seen from the literature that faults occurring in sensor nodes are unrelated stochastically but their measurements are spatially correlated [43]. We take two sensor nodes denoted $V_{i}$ and $V_{j}$, which are both neighbors. We further suppose that $X_{i}$ and $X_{j}$ are the sensor readings reported by $V_{i}$ and $V_{j}$, respectively. Both have the same readings when $\left(\left|X_{i}-X_{j}\right|<S\right)$, where *S* depends on the application. For example, in monitoring bolt loosening, two neighbour sensor nodes have the same voltage. Similarly, in temperature monitoring, two neighbouring sensor nodes are observed to have the same reading. Therefore, *S* would be a small number. Moreover, in the event detection applications, the decision taken at sensor nodes is sent to neighbor nodes, so in these cases *S* is set to be 0. Generally, two types of faults are detected while performing fault diagnosis operations in a network: permanent faults and soft faults.The following subsection describes how to address both.

### 2.5. Permanent Faults and Soft Faults

**Figure 5:**
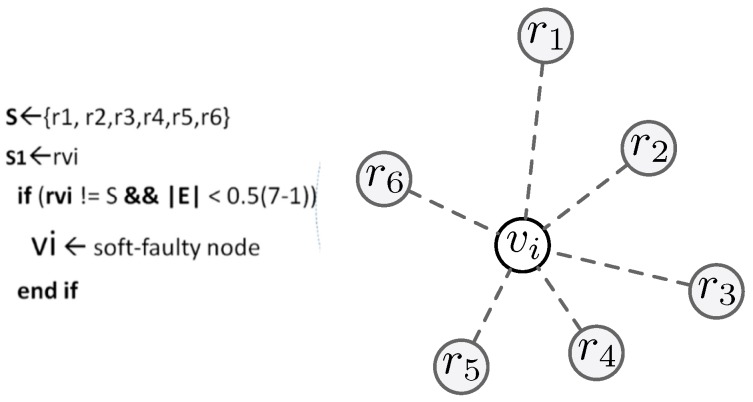
Fault detection process.

In WSNs, permanent faults are detected by algorithms using a timeout mechanism. Each node maintains a neighbor table of one-hop nodes N(·) In Figure 5, a node $V_{i}$ declares $V_{j}$ as hard faulty if it does not listen to its reading before timeout $\left(T_{\mathrm{out}}\right)$. The value of $\left(T_{\mathrm{out}}\right)$ should be chosen carefully. All of the nodes in the neighboring set of $V_{i}$ could reply to it before the mentioned time. On the contrary, the soft faults can be detected by the following method. Upon receiving sensor readings, the node $V_{i}$ forms a set of one-hop neighboring nodes; that is, those having the same reading, such as $\left\{E\right\}\in N(V_{i})$. If node $V_{i}$’s reading does not agree with the reading of *S*, and the cardinality of set *E* is less than a threshold (Θ), then $V_{i}$ is said to be a soft faulty node. The optimal value of Θ is obtained by using 0.5(*N*-1), where *N* is the number of neighboring nodes in one hop [44]. Out of the many types of faults, intermittent faults are the most important and the most difficult to monitor as well as detect.

### 2.6. Detection of Intermittent Faults

**Figure 6:**
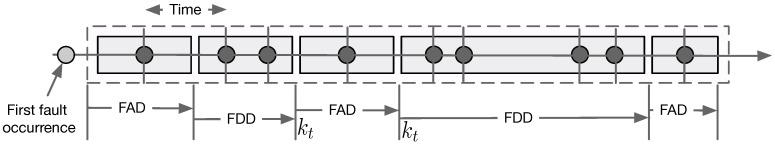
Fault detection process.

In the case of intermittent faults, once it is observed that an intermittent fault has occurred in a node, the node starts noticing the fault for a length of time until it reappears again; this length of time is called the fault appearance duration (FAD). The errors that reappear due to permanent faults or correlated intermittent faults reappear after what is called the fault disappearance duration (FDD) (see Figure 6). Intermittent faults are further categorised by considering their measurements, and then estimating their disappearance and reappearance at discrete time intervals, such as $k_{T}$ for $k_{T}=1,2,\ldots ,k_{\max}$ (see Figure 6).

**Figure 7:**
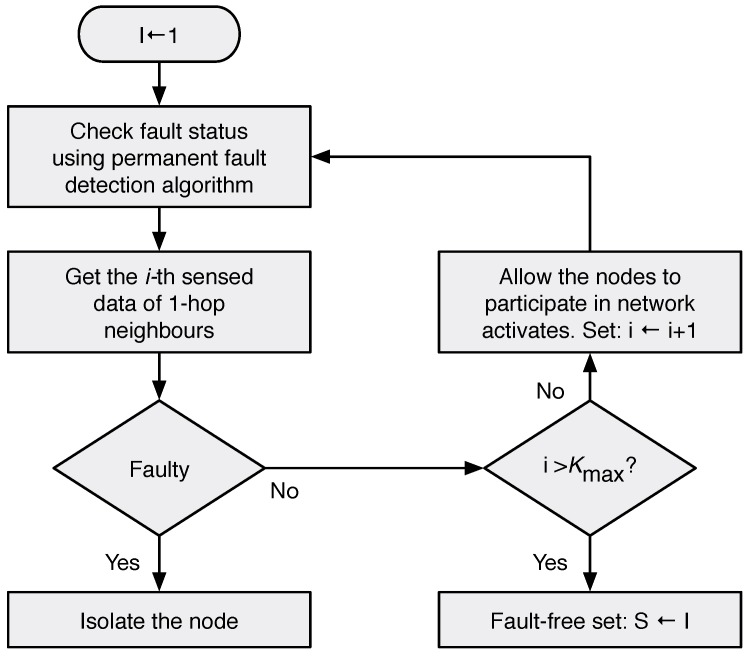
Fault detection flowchart.

Intermittent faults are detected using a permanent fault detection algorithm at discrete time intervals $k_{T}$. Figure 7 presents a flowchart of the algorithm, which is used by each node to collect the observations regarding fault states of all of the nodes via local views. At first, the value of the counter is set to 1 using the algorithm node that decides its own fault status. The faulty label in the conditional block of Figure 7 presents a snapshot view of such a diagnostic round. The algorithm executes as long as no error is detected or it reaches the maximum values of $k_{\max}$.

## 3. Related Work

**Figure 8:**
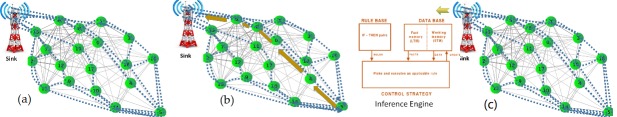
Fault detection approaches: (**a**) proactive; (**b**) active and (**c**) passive.

In WSNs, fault diagnosis is performed by analysing anomalies in the data. Generally, three detection approaches are used in a network for data collection: proactive, passive and active (see Figure 8). In proactive approaches, debugging agents are fixed on each node in the network. These agents gather status information periodically and then send it to the sink for evaluation. In passive approaches, the network is monitored continuously and status information regarding each node is reported continuously. However, this monitoring imposes extra computation cost and traffic overhead on WSNs. In active approaches, the sink sends a desired query into the network wirelessly. The query is received by all of the nodes in its proximity, but only those nodes that have been asked for reply. In WSNs, all of the aforementioned techniques are practised by considering the type and nature of the application. The following subsections will explain these techniques by drawing on the supporting literature. They will also explain which type appears to be more energy efficient, which accurately observes symptoms, which is efficient with respect to time, which is more cost effective in terms of network traffic and which effectively monitors network topology during fault diagnosis. We present each approach in detail to illuminate both the advantages and the limitations.

### 3.1. Proactive Approaches

According to the fault diagnosis literature, most of the schemes are based on proactive approaches [32,45,46,47,48,49,50]. They are embedded in the nodes to perform fault diagnosis and they periodically scan the health of the nodes (see Figure 8a). These approaches provide a high volume of information about the nodes’ residual energy status [51] or a list of neighboring nodes [52]. This subsection will consider some of the proactive schemes that are used to diagnose faults in networks.

Felemban et al. [53] proposed a probabilistic scheme that can be used to guarantee Quality-of-Services (QoS) in WSNs. It has the improved timeliness and reliability features that are required for such networks. Ji et al. [54] suggested a scheduling algorithm that is based on the multi-path concept. This scheme enhances the data collection process because it collects data using single radio multichannel WSNs.

Ping et al. [55] presented the time synchronisation issue in small networking devices and they also proposed a technique applicable to both single- and multi-hop WSNs, which they called delay measure time synchronisation (DMTS). This scheme requires *N* number of total messages to be exchanged to synchronise the entire network. This application is available in TinyOS as service. Isabel et al. [56] devised a general formula that can be used to calculate the lifetime of WSNs and is independent of the network architecture and protocol, initiation of data collection, channel fading features, lifetime definition and energy depletion model. Based on the derived formula, the medium access control (MAC) protocol is used to obtain both the channel state information (CSI) and the remaining energy status of the sensor nodes.

Zhiyang et al. [57] presented a fault diagnosis model that is based on [58,59,60]. In this model, each node forms a cluster with its neighboring nodes. A node is called a neighbor of any node if it is in its transition range. The diagnostic operation is periodically performed within the cluster by comparing the result with its neighboring nodes. The diagnostic protocol is divided into the following five steps:

**Step** **1** *Starting a diagnosis session:* A diagnosis session is started periodically or when abnormal behavior is detected. A node sends a TestReq message to its neighbor node when the session is started and starts two timers.
**Step** **2** *Test:* After receiving the message, each node sends its own TestReq message and starts its two timers. It then broadcasts the result to its neighboring nodes through a TestRes message. The test case results should be identical at each node after execution. Moreover, functional errors are tested using test cases related to the function of the nodes.
**Step** **3** *Comparison Phase 1:* When a node receives a TestRes message, it stores it. Once it receives a Timer1 message, it compares the result to the test results of its neighboring nodes. If more than *K* (where *k* is set to 2) matches are found, then it declares itself fault-free and sends an IAmOk1 message to its neighbors. Furthermore, after data transmission, the remaining harvested energy is stored in the energy-storage device to recharge up to the maximum storage capacity $E_{\mathrm{capacity}}$. Algorithm 2 presents the above steps.
**Step** **4** *Comparison Phase 2:* In case a node does not recognise itself as a fault-free node in the last step but it receives an IamOk1 message from neighboring nodes, it compares its own test result to the test results performed by the IAmOk1 message. If its finds that these results are equal, then it recognises itself as fault-free and sends an IAmOk1 message to the neighboring nodes.
**Step** **5** *Dissemination:* After receiving the message IAmOk1 or IAmOk2, it forwards the message to its neighboring nodes.

The diagnosis session ends after receiving a Timer2 message from the node. The pseudo-code of the protocol is shown in Algorithm 1. In the following code, 1-lb is used to send a message to its neighbors. The protocol distinguishes itself from existing approaches by two advantages. First, it does not have any embedded agent that sends the status information to the sink and it also works in a local area, such as a node and its neighboring nodes. It is claimed that it does not put much computational cost on the sensor nodes or impose a large communication overhead on the network. Second, it is a proactive protocol and is suitable for applications that do not have continuous connections. Harte et al. [61] introduced a novel technique for the detection of faults in WSNs, which uses a tree-based heuristic reasoning technique. It infers the cause of faults by diagnosing the state of links and nodes, as well as other factors involved in optimising the recommendation of the most effective status information collected in real time. The problem with this method is that it uses a periodic sampling method that overloads the network under heavy traffic load.
**Algorithm 1:** Demonstration of fault diagnosis protocol ${}^{1}$.  
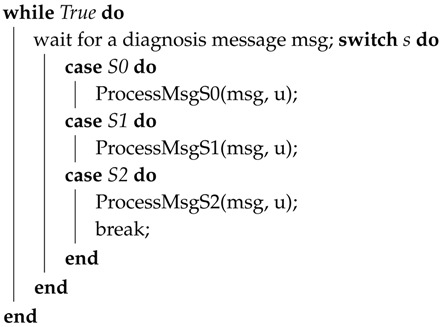
^1^ Details of rocessMsgS0, rocessMsgS1 and rocessMsgS2 can be found in Algorithms 2, 3 and 4 respectively.

Zhao et al. [62] presented a dynamic-model-based fault detection scheme, which uses the recurrent neural network (RNN) technique for fault detection and classification in sensor networks. Although this method achieves better efficiency compared to the Kalman filtering algorithm [63], it does not have better accuracy. Mahaptro et al. [64] proposed a cluster-based fault diagnosis (CDFD) algorithm, which considers the possibility of fault and communication situations used at each sensor node. It uses the sensor nodes’ special correlation measurements to determine the local diagnostic view. The limitation of this algorithm is its accuracy, which is not good because it may consider some faulty nodes as fault-free.
**Algorithm 2:** Procedure: ProcessMsgS0 (msg, u).  
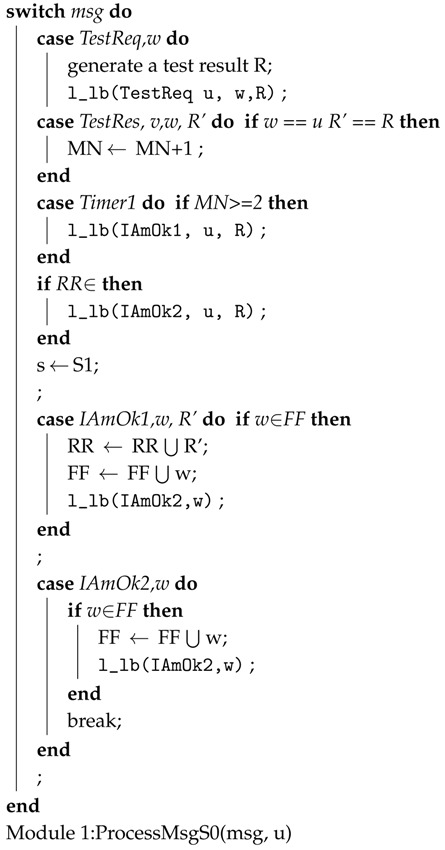


Banerjee et al. [65] suggested improving the performance of a network by effectively handling the faulty sensor nodes. They identified a sensor circuit’s fault using a vector-based fault detection model. The performance of this protocol is better in terms of lifespan, network coverage and energy utilisation. In addition, it manages the scalability of the network using efficient fault detection and routing (EFDR) algorithms. This algorithm handles the faulty nodes effectively and efficiently. Performance evaluation shows an 85% improvement over competing protocols.

Moreover, the algorithm in [66] identifies nodes with data faults. It considers a node to be faulty based on both the measurement of and the distance of the node from the event. A node is said to be faulty if a significant mismatch is found between the ranks of the sensed data and if reading the collected data violates the distance monotonically.
**Algorithm 3:** Procedure: ProcessMsgS1(msg, u).  
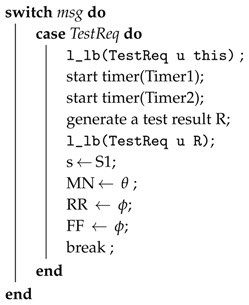

**Algorithm 4:** Procedure: ProcessMsgS2.  
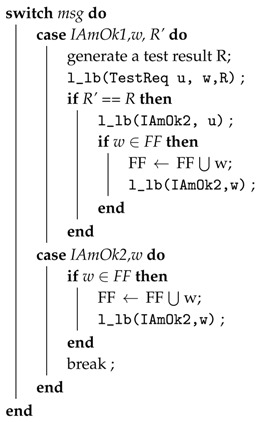


Generally, these approaches have several limitations; for example, their deployment involves difficulties such as rapid energy depletion, packet loss and high end-to-end delay. To address these problems, network diagnostic tools are used in the control environments. Given that constraint-oriented WSNs are deployed in harsh and inaccessible environments, these networks are, therefore, difficult to access from the outside once deployed. These approaches also impose high traffic overhead on the resources of constraint-based networks. Furthermore, they also face the loss of status information. Passive approaches are used to address these issues.

### 3.2. Passive Approaches

The proposed passive schemes consume less network resources, such as processing, computation, communication, low-bandwidth utilisation and low traffic overhead compared to the proactive approaches. These schemes do not collect special data for the process of fault diagnosis in a network; instead, they perform the process on normal data packets. Additionally, some schemes use model-based techniques (such as Artificial Neural Networks (ANN), rough theory, etc.) to diagnose and detect faults in networks. Techniques based on these models have been an object of focus since 1971. They were originated by Beard [67], who used analytical redundancy instead of hardware redundancy. The outcomes of these techniques have been published in several books [68,69,70,71]. Using these models, fault diagnosis algorithms can be developed that consider the consistency between expected and actual outputs. This subsection describes the passive schemes that have been developed based on these models to perform fault diagnosis.

Debugging tools are difficult to integrate with programs. It would be even worse to implant proactive information-collecting schemes in a network because they would increase the depletion of energy and hastily reduce the overall lifetime of the network. Pan et al. and Zhang et al. [72,73] recommended online diagnosis because it is lightweight with respect to the network and to monitoring network symptoms for the sink. This technique uses a probabilistic inference model to effectively detect and deduce the root cause of abnormal behavior occurring in the network. Compared to proactive approaches, passive approaches use normal data packets for back-end fault diagnosis. These approaches can be maintained in systems at low cost. It is also believed that the applications respond in a timely fashion without affecting network performance. Several important passive approach schemes that are described in the literature will be discussed next.

According to the descriptions in [74,75], inference-based network diagnosis methods have been most widely focused on and used in enterprise networks for inferring the root cause of service failures. Three types of inference models are available: deterministic, non-deterministic and hybrid. These types of methods have been considered to determine the root cause of abnormalities observed during monitoring. A deterministic model uses numbers as inputs and produces numbers as outputs. The number of results produced is fixed, irrespective of how many times the experiments are repeated. The model calculates the same output(s) if the input provided to the model remains fixed. In the non-deterministic model, the probabilistic distribution is determined by performing multiple runs. It contains some inherent randomness. In this model, the same set of inputs or conditions could lead to different outputs. This is also called black box modeling. Finally, the hybrid model mixes both deterministic and non-deterministic models. It determines how things are known and the impact of the uncertainties existing in the inputs on the output of the model. Those components that do not contribute much to the variation of results can be considered to be deterministic. Furthermore, those components that contribute significantly are treated as probabilistic. This results in a hybrid model [76]. Since these model-based techniques are beneficial and advantageous, they have been used to perform the passive fault diagnosis in WSNs.

Liu et al. [28] introduced a probabilistic diagnosis algorithm that performs fault diagnosis by using a probabilistic model on network status parameters. However, this model is inefficient and complicated. Nie et al. [77] suggested an algorithm for fault diagnosis, which they called diagnosis based on sending data (DSD). However, the DSD algorithm does not consider the time-domain impact. Mio et al. [78] proposed an agnostic diagnosis (AD) algorithm, which is a lightweight fault diagnosis algorithm. Although it is useful for static fault diagnosis, it is not recommended for dynamic fault diagnosis. Yunhao et al. [79] advanced the nucleus network management systems (NMS), which exports specific metrics but does not analyse them.

**Figure 9:**
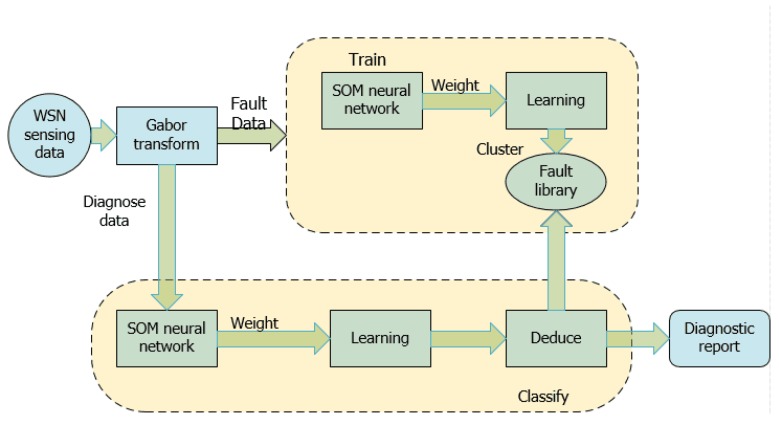
Passive approach: Process of inference model following [80].

Lufeng et al. [80] presented an algorithm that is based on the time-domain features of sensing data (TDSD). The TDSD algorithm is a solution of the problems mentioned in [28,77,78]; it is also a passive algorithm. When troubleshooting a WSN, it uses the domain characteristic of perception data to detect and classify faults in a network. In addition, this algorithm has been combined with the discrete Gabor transformation and SOM neural network technology, and thus it performs fault detection and classifications effectively (see Figure 9).

### 3.3. Active Approaches

Currently, in active approaches sensor node debugging is performed using a combination of simulation, visualisation tools, log file interrogation, passive monitoring and tracing programs. Simulations [81,82] help to reduce cost, the length of the deployment cycle, and thereafter reduce the need to repeat the experiments as many times as possible. Nevertheless, it does not replace real hardware requirements, such as real-time network dynamics, environment dynamics, other hardware details, MAC and numerous timings. Log files can obfuscate important events because they capture much unfiltered data. Visualisation tools help to perform real-time information debugging but they do not identify failures. For example, EmStar visualiser [83,84] collects the historical data for future reference.

Although these tools can identify link quality and neighbour-level connectivity, there is a conflict between these properties because it is possible that the node has no neighbour despite having a high-quality link.

Ramanatham et al. [26] proposed a protocol, called Sympathy, which is the combination of all the above-mentioned aspects. It collects information from the sensor nodes at runtime, such as a routing table and flow information, to judge the possibility of a fault while maintaining awareness of network exceptions. It provides several command-line and web-accessible mechanisms through which a user can query and control the system. Sympathy provides a ping-about command that flows into the network to ask for information about the nodes. When a node receives a query, it replies with available standard metric packets. In addition, all of the other nodes that have the same information regarding the query requested from the nodes, such as number of packets communicated with, or the time of last communication, transmit the data back to the sink.

Sympathy’s web interface displays the number of nodes graphically. It also allows the user to query any node to retrieve the requested information and highlight the failure. Performance evaluations show that Sympathy detects the injected network failure accurately with relatively low latency and minimises network traffic. It also integrates a graphical interface that can visualise metrics and environmental data [85].

Jiang et al. [86] proposed an active fault diagnosis algorithm that actively gathers information based on a random walk approach. It uses a compressive sensing approach to deal with the signal for a space information network (SIN). A SIN is different from WSNs in that a SIN possesses a long time delay and inconsistent topology. Therefore, existing fault diagnosis techniques cannot be applied to SINs. Performance evaluations show that Jiang et al.’s [86] algorithm can handle the problem much more effectively under SIN circumstances. Tolle et al. [87] suggested that a sensor network application that uses the nucleus management system (NMS) infrastructure. It exports debugging and monitoring information, and sends application metrics. This infrastructure supports the export of counters and statistics that is both easy-to-use and lightweight in terms of network overhead. Zhao et al. [88,89] presented an energy-efficient method that continuously computes the in-network processing calculations, such as sum, average, the number of packets lost, energy levels and the number of packets that help in debugging.

Kim et al. [90] demonstrated the transmission of network metrics from the nodes to the sink; however, this is not a novel idea. In evaluation scenarios, when network traffic increases, the data cannot reach the destination within the specified time. A technique, called Mint-route, includes periodic sending of neighbour tables. It also helps in performing debugging operations at the sink, and is used to avoid network traffic congestion and help minimise delay. Ruan et al. [91] proposed the DeBox system, which is motivated by Sympathy’s design (as discussed above). Since it exposes minimum states to an application in real time, DeBox exhibits better performance and tuning than passive schemes that give information *post facto*. Sympathy does not consider performance; it concentrates only on fault detection and debugging, and it enhances a system’s transparency and visibility.

## 4. Summary of the Main Contributions and Important Terminology

This section has two purposes. First, to provide a succinct overview of the techniques used in each fault detection approach category to present a big picture of the trends for performing fault diagnosis in WSNs (see Table 1); Second, to present important and confusing terms of the field used and, in addition, to help readers easily understand the main concepts described (see Table 2).

## 5. Open Research Challenges

WSNs have steadily become a cutting-edge technology of the 21st century for the development of wireless sensor applications. Moreover, WSN research is a very important area of study because its applications are prevalent in almost all walks of life. Owing to this importance, much work has been performed in the last decade. Nevertheless, WSN research still requires more work to fulfill the requirements of current applications development. The following list of open research challenges and issues should be addressed in future fault diagnosis schemes:

- Intelligent techniques are required to minimize network overhead and bandwidth consumption. Moreover, there is a strong demand to perform online fault diagnosis by considering the constraints on the nodes, networks and environments. A protocol needs to be devised for the efficient load balancing and recovery of faulty sensor nodes, especially in the case of multimedia sensor nodes. More energy-efficient protocols should be developed by analyzing end-to-end transmission time delay, by minimizing overlap among the transmission ranges and by reducing the computational cost on individual sensor nodes.
- Safer and more reliable algorithms should be developed because of the increasing demands of dynamic systems.
- An optimal technique is required to identify crashed (faulty) nodes in those application scenarios in which battery replacement is feasible or is otherwise energy efficient in WSNs.
- There is a strong demand for a reliable fault diagnostic protocol with low latency. Given that WSNs are increasingly being deployed to monitor critical conditions (such as toxic gas leakage, fire and explosions), a fast, reliable and fault-tolerant algorithm should be developed that can help in all three approaches; that is, query-based, periodic or event-driven. In this case, even if an emergency occurs and nodes fail or a path is disrupted, data are still delivered to the sink.
- Fault diagnosis techniques are required to be used in nonlinear, uncertain systems based on processing modelling.
- Reliability and scalability is required to maintain a better QoS in large-scale sensor networks, which is a major challenge that demands the development of a protocol to achieve a better QoS; for instance, through sensor consensus, interaction between different types of sensors, harsh environments and electronics.

## 6. Conclusions

WSNs have proven to be one of the cutting-edge technologies of the 21st century for developing low-power wireless applications for pervasive computing. Since such networks are normally deployed in remote, harsh and inaccessible environments, and must work autonomously without any manager or operator, nodes in WSNs are only at the disposal of the chosen network protocol in any given situation. In addition, nodes have limited resources, such as energy, storage and computation power, and can become faulty very easily. In cases in which such a network does not feature fault diagnosis, then not only might limited resources be consumed or exhausted but the network lifetime might also be shortened. Consequently, the importance and implications of fault diagnosis on the network cannot be ignored. Thus, this paper presents a survey of WSN fault diagnosis from an operational perspective and divides the proposed schemes into three different approaches: (a) proactive; (b) passive and (c) active. In this paper, each approach is explored by reviewing supported schemes available in the extant literature, and presenting their advantages and limitations with respect to each other. Table 1 briefly lists the proposed schemes and related research trends. Such a survey not only helps researchers understand the current scope of fault diagnosis techniques from a network perspective available in the extant literature, but can also inform the design of more efficient, reliable, effective, scalable and robust fault diagnosis techniques in the future. Finally, we outline the open research challenges and issues with the aim of providing some direction for proposals of more desirable protocols that could meet the requirements of current and future applications.

## Figures and Tables

**Table 1:** Overview of Fault Diagnosis Techniques.

Author(s)	Year	Technique Used	Short Description
Proactive Techniques			
Ping et al. [55]	2003	Delay measurement time synchronisation (DMTS)	Flexible, lightweight and applicable on both single- and multi-hop-based networks. Takes only one clock click to synchronise nodes available in single hops and uses *n* time message exchanges to synchronise the entire network. Thus, DMTS minimises the network traffic and is also considered as more energy efficient because radio communication is a significant source of energy consumption.
F. Felemban et al. [53]	2005	Probabilistic method	Suggested for improving QoS, timeliness and reliability in WSNs.
Elhadef et al. [59]	2006	Probabilistic fault model	Uses several important distributions, such as Bernoulli failure distribution, gamma failure distribution and exponential failure distribution, to determine the local and global performance of the proposed scheme. Performance evaluation shows that it determines fault-free nodes successfully, even when the percentage of fault-free nodes is less than 50%.
You et al. [57]	2011	Probabilistic fault model and probabilistic analysis	Suggests modeling of diagnostic algorithm operating in a cluster of nodes using probabilistic analysis of the local and global performance.
Shouling et al. [54]	2011	Multi-path scheduling algorithm	Introduces the capacity of continuous data collection in dual radio multi-channel.
Cheng et al. [12]	2012	Probabilistic diagnostic algorithm	Analyses negative binomial failure distribution under fault clustering. Tested against wafers. A simple structure is given as a test case to determine the status of each die. Efficient because it performs tests on all dies in parallel and, hence, saves a lot of time in finding the die through probe testing.
Mouradian et al. [46]	2013	Probabilistic nature of radio link	Achieves reliability by using the probabilistic nature of the radio link. Suggests a theoretical framework based on a reference model for two types of routing schemes, such as unicast and broadcast based.
Banerjee et al. [65]	2014	Vector-based fault detection model	Identifies sensor circuit fault identification using vector-based fault detection model. Performance of this protocol is better in terms of lifespan, network coverage and energy utilisation.
Zhao et al. [88]	2014	Abstracted scan method	Helps users to be informed regarding the resources and applications running on the sensor nodes and helps manage sensor node activities accordingly. Additionally, performs in-network aggregation to form abstracted scans of the nodes. Specifically, it proposes the development of a residual energy scan to determine the remaining energy distribution in the network.
Mahapatro et al. [64]	2014	UCR and two-state Markov model	CDFD performs online fault diagnosis by using the spatial correlation in a two-state Markov model for the good approximation of slow and fast fading, and integrates it with an unequal cluster-based routing (UCR) protocol. Without considering wireless channel impairments, it identifies both soft and hard faults. Additionally, does not impose any traffic overhead and the diagnostic messages are conveyed using routine network traffic.
Gupta et al. [47]	2014	Top-*K* interesting subgraph discovery	Focuses on the following two challenges:. First, it introduces a two-index structure, such as a topology index and a graph-based maximum meta-path weight index, which are both calculated offline. Second, it suggests novel top-*K* mechanisms to use these indexes to efficiently answer the query online.
Proactive Techniques			
Hayes et al. [45]	2015	Single-hop or blind forwarding	This algorithm, PHASeR, uses robust and dynamic data routing towards the sink in mobile environments. Uses single-hop count metric or blind forwarding method to send the messages through a multi-path in the network. PHASeR is analysed mathematically on average packet delivery, throughput and packet delivery ratio. It is then evaluated against mobility, scalability and traffic loads. Recommended for a wide variety of emerging applications.
Chanak et al.	2016	Undirected graph	The main objective of proposed technique is to overcome a network failure condition in a WSN in an energy efficient manner and relay data packets from the source nodes to the BS with minimum time delay. The network conditions can effect the QoS. The network is believed to be tolerated from these failures during the data routing stage then QoS of the WSN can be maintained.
Active Techniques			
Tolle et al. [87]	2004	Nucleus management systems	Nucleus management system (NMS) infrastructure exports debugging and monitoring information.
Ramanathan et al. [26]	2005	Debugging tool called Sympathy	Collects information about link quality or neighbour-level connectivity from sensor nodes at runtime.
Kim et al. [90]	2011	Mint-route technique	Suggests debugging operations to be performed at the sink node.
Ruan et al. [91]	2011	Sympathy-based approach	Performs fault detection and debugging based on the Sympathy approach. In addition, it enhances a system’s transparency and visibility.
Liu et al. [79]	2013	Event-based routing structure	Carried out on an event-based routing structure in GreenOrb. GreeenOrb consists of 330 nodes and is deployed to monitor forests. Adapts to the wild environment smoothly and is an excellent platform for observing large-scale sensor networks.
Jiang et al. [86]	2015	Random walk approach	Actively gathers information based on the random walk approach. It also uses a compressive sensing approach to deal with the single random walk approach.
Gao et al. [92]	2015	Real-time monitoring and fault tolerance	Performs real-time monitoring, diagnosis and fault tolerance. Has the potential to become an emerging research direction for real-time fault-tolerance control and applications.
Geest et al. [2]	2015	Analytical models	Suggest a simple fault detector based on the difference of synchronous detection of the machine and inverter neutral voltage, which is suitable for hardware implementation and can function as an independent observer of a drive system.
Wang et al. [4]	2015	Lyapunov function	Suggests a robust, adaptive fault-tolerance consensus protocol for multi-agent systems to address unknown nonlinear dynamics and unexpected actuator faults. Although it does not directly depend on the diagnosis of the faults, it does depend on the compensation of its ultimate impact; such an impact has been reflected in part of the lumped uncertainties in the system.
Passive Techniques			
Chen et al. [68]	2000	Model-based technique	Based on increasing demand of dynamic systems, which insists that the systems be made more reliable and safe. Focuses on the subject of fault detection and isolation requiring more attention to become an established field of research in control engineering. Provides comprehensive material on model-based fault detection isolation (FDI).
Nie et al. [77]	2012	Inference model	Determines the root cause of failures by finding the relationship between the sensed data and the failures that occurred in the network without adding any additional traffic overhead. Saves them the need for a knowledge library to take the decision instead of focusing on collecting diagnosis metrics that impose heavy traffic overhead on the network.
Seydou et al. [93]	2013	Model-based techniques	Based on a fault detection model for a particular class of nonlinear systems, called flat systems, to address an original solution for a flat system in actuator fault diagnosis.
Liu et al. [28]	2013	Probabilistic inference model	Does not incur additional traffic overhead for the collection of desired information. Uses a probabilistic inference model for online diagnosis of an operational WSN, which encodes dependencies existing among different network elements.
Zhang et al. [73]	2015	Classification framework	Suggests a fault detection framework from the perspective of energy efficiency subject to facilitating the fault detection methods and the evaluation of their energy efficiency. A classification of fault detection approaches is provided using the same framework, which is based on several characteristics, such as energy efficiency, correlation method, evolution method and detection accuracy.

**Table 2:** Brief overview of important fault diagnosis terms.

Term	Definition
Deterministic model	Uses numbers as inputs and numbers as outputs, and assumes that its outcome is certain if the inputs of the model are fixed. The output of the model is fully determined by the parameter values and the initial conditions. It provides same result for same input.
Non-deterministic model	Possesses some inherent randomness. The same set of parameter values and initial conditions will lead to the assembly of different outputs. Also called black-box modeling. It, on the other hand, exhibits different result for same input.
Stochastic model	According to probability theory, in this model the values of the parameters, measurements, expected inputs and disturbances are unpredictable because of a random variable. Thus, it can be classified as a non-deterministic model because of its random nature. This model is more informative than deterministic model due to uncertaity in varying behavioral characteristics.
Probabilistic model	Incorporates random variables and probability distributions into the model of an event or phenomenon and observes the system and gathers its statistics before performing any action. It estimates on the basis of historical data.
Hybrid model	Mixes aspects of two or more models; that is, some parameters of the deterministic model are randomly defined according to experimental observations.
Offline debugging	Normally preferred in in-situ network diagnosis and carried out when a failure has occurred due to sensor behavior and not strictly controlled network scale.
Online debugging	Starts dealing with the failure at runtime by rapid verification closure with capability to execute the design back and forward.
Active diagnosis	Injects some queries or probes into the network and determines or infers the quality of the network’s performance through measurement parameters.
Passive diagnosis	Does not collect special data for fault diagnosis in the network [87].
Online fault diagnosis	Finds faults during system runtime.
Offline fault diagnosis	Collects data of the system states so that it can later perform fault analysis. Also called *post-mortem*.
Fault Diagnosis	Consists of detection, isolation, identification and recovery [83].
Fault	An unusual change in, or deviation from, one or more characteristics of a system’s standard, acceptable or usual conditions [38].
Failure	An omission in occurrence, performance, performing duty, expected actions, achieving goals or achieving objectives as prescribed.
Error	A system state that may cause a subsequent failure: a failure occurs when an error reaches the service interface and alters the service. A fault is the adjudged or hypothesised cause of an error.
Incipient fault	Method for early detection of soft faults.
Fault Identification	Detects whether the node is faulty or free-faulty, such as fault recovery protocol.
Fault Recovery	After failure detection, the recovery process is started to efficiently recover from a failure.
Fault Isolation	Removing the faulty nodes from the network after identification and verification.
Local diagnostic view	This diagnostic is created by the combination of nodes in a network.
Global diagnostic view	This diagnostic is created by the sink
Special correlation	To achieve satisfactory coverage, spatially dense sensor deployment is preferred in WSNs. Consequently, many nodes record the same information about an event. Thus, spatial correlation increases with the degree of density or decreasing inter-node separation.
Temporal correlation	Degree of correlation between two consecutive measurements that may vary due to the temporal variation in features of the phenomenon. Its computation fluctuates with respect to time of the data, such as time series.
Spatio-temporal correlation	A combination of both spatial and temporal features that brings significant advantages to the design of energy-efficient communication protocols for WSNs.
Static fault diagnosis	In static models, the diagnosis problem is formulated as one of maximizing the posterior probability of component states given the observed fail or pass outcomes of tests. It works under supervised learning mechanisms such as ANN.
Dynamic fault diagnosis	In the context of dynamic models, states components are to be evolved as independent model, in such model, at each time epoch, we have access to some of the observed test outcomes. Given the observed test outcomes at different time epochs, the goal is to determine the most likely evolution of the states of components over time. It operates under unsupervised learning techniques such as Independent Markov Chain Model.
Hardware redundancy	Consists of replication of computers, sensors, actuators and other components, and is used to achieve the mechanism of FDI.
Analytical redundancy	Also known as functional redundancy; based on mathematical model of the system being monitored.
Model-based fault diagnosis	It detects soft faults, such digital controller, digital filter, as as well as hardware faults, such as defective construction, actuator faults, sensor faults, abnormal parameters, external obstacles (collision, clogging). It performs the following three important tasks: (i) fault detection; (ii) fault isolation; and (iii) fault identification or analysis.
Robust fault detection	Capable of predicting soft, small or early faults in a system’s components before being caught by a human operator or automation system.
Online detection	Real-time detection [93].
